# Case Report and Literature Review: Primary Pulmonary NUT-Midline Carcinoma

**DOI:** 10.3389/fonc.2021.700781

**Published:** 2021-08-30

**Authors:** Yunxiang Zhang, Kai Han, Xiaotong Dong, Qian Hou, Tianbao Li, Li Li, Gengyin Zhou, Xia Liu, Guifeng Zhao, Wei Li

**Affiliations:** ^1^Pathology Department, Weifang People’s Hospital, Weifang, China; ^2^Scientific Research Department, Qingdao Geneis Institute of Big Data Mining and Precision Medicine, Qingdao, China; ^3^Pathology Department, Qilu Hospital of Shandong University, Jinan, China; ^4^Ophthalmology Department, Affiliated Hospital of Weifang Medical University, Weifang, China; ^5^Prenatal Diagnosis Department, Weifang People’s Hospital, Weifang, China; ^6^Thoracic Surgery Department, Weifang People’s Hospital, Weifang, China

**Keywords:** bromodomain-containing protein 4 (BRAD4), lung, genomic analysis, nuclear protein of the testis (NUT), NUT-midline carcinoma (NMC)

## Abstract

Nuclear protein of the testis (NUT) carcinoma is a very rare and aggressive carcinoma characterized by chromosomal rearrangement. NUT-midline carcinoma (NMC) can occur anywhere in the body, but most of the tumors are found in the midline anatomic structure or mediastinum. Pulmonary-originated NMC is extremely rare and often difficult to be distinguished from other poorly differentiated tumors, making the diagnosis awfully challenged in clinical practice. There are less than 100 cases of NUT carcinoma reported so far. In this study, the diagnosis and molecular mechanisms of reported NUT carcinoma cases were reviewed. Furthermore, a case of primary pulmonary NUT-midline carcinoma and its pathological features was reported. The process of pathological identification and genomic analysis for establishing the diagnosis was discussed. We found that NUT carcinoma could be identified by combining CT, H&E staining, immunohistochemistry (IHC), and molecular tests. The development of NUT carcinoma might be associated with mutation of *MYC*, *p63*, and *MED24* genes and the Wnt, MAPK, and PI3K signaling pathways. Our study provided a detailed molecular mechanistic review on NMC and established a procedure to identify pulmonary NMC.

## Introduction

Nuclear protein of the testis (NUT) carcinoma is a very rare and aggressive carcinoma characterized by chromosomal rearrangement. NUT-midline carcinoma (NMC) was originally named proline-rich undifferentiated carcinoma (PRUC) because of the genetic build-up of proline (1). NMC is an aggressive malignant epithelial neoplasia and its average survival is less than 1 year. NMC is characterized by rearrangement on the NUT gene, which is located on chromosome 15q14, resulting in the bromodomain-containing protein 4 (BRAD4)-NUT fusion oncogene. NMC primarily occurs in children and young adults. In the USA, the first case was reported in a 12-year-old girl with an epiglottis mass. She did not respond to antibiotics treatment and died shortly due to tumor recurrence that closed her airway. This tumor is usually diagnosed by positive karyotyping for t(15;19) and confirmed by fluorescence *in situ* hybridization (FISH) (2). The histological feature cannot be used as diagnostic criteria since the morphology is a poorly differentiated carcinoma and tends to be pleomorphic. NMC is considered a genetically defined disease and does not arise from a specific organ. Most of them are found in midline anatomic structure and mediastinum, within the upper aerodigestive tract and thymus. Some NMCs might arise in bone (3), bladder (4), abdominal retroperitoneum (5), pancreas, and salivary glands (5, 6), and pulmonary origin NMC is extremely rare.

Pulmonary NMC is classified as a type of novel disease at the WHO lung cancers. This carcinoma was recognized in the thymus in 2004 (7). At present, literatures about pulmonary NMC are rare. Tanaka et al. described only two possible pediatric cases of pulmonary NMC in their 41-year investigation (8). Haruki et al. screened 128 lung cancer tissues using fluorescence *in situ* hybridization, but none of the screened tumors showed t(15;19) (9). Sholl et al. reported eight cases of primary pulmonary NMC and used NUT immunohistochemical screening. The result suggested that although primary pulmonary NMC is rare, it has highly clinical, imaging, and pathological features (10). NMC is often undiagnosed or misdiagnosed due to a lack of comprehensive knowledge of NMC and the lack of reagents and expertise needed to diagnose the disease. A case previously misdiagnosed as Ewing’s sarcoma (ES) (11)/primitive neuroectodermal tumor (PNET) (12) was reported, which was later confirmed as NUT carcinoma by next generation sequencing (NGS). Stelow et al. investigated the incidence and expression of NUT rearrangement in a series of undifferentiated carcinomas of the upper aerodigestive tract (UCUAT) in the diagnostic significance and described the histological characteristics of these tumors in detail (13). Hellquist et al. used immunohistochemistry, FISH, and reverse transcription-polymerase chain reaction (RT-PCR) to observe NUT rearrangement to diagnose NMC. They found three previously published cases and added the 4th one of their own (14). Bishop et al. identified all NMC specimens (confirmed by molecular testing and/or NUT immunoreactivity) in two academic centers and identified 26 NMC cytopathological samples from 13 patients. The results showed that the cytological characteristics of NMC overlapped with other tumors to a certain extent (15). Recently, a rare case of NMC in an 8-year-old Turkish boy was reported by immunoreactivity to nut antibody (FISH), suggesting that NMC should be considered in the differential diagnosis of undifferentiated carcinoma located in the midline (16).

So far, only a small number of case series and rare case reports have described the cytopathological features of NMC. Pulmonary-originated NMC is rare and often difficult to identify from other poorly differentiated tumors. Thereby, it is a challenge to make an accurate diagnosis. In this study, we investigate the pathological feature of primary pulmonary NMC and report a case of a 16-year-old boy with a growing pulmonary mass in the upper lobe of his right lung. The tumor is diagnosed as pulmonary NMC by immune-histological examination and qPCR-assisted assay.

### Diagnosis and Molecular Mechanism of NMC

**Figure 1:**
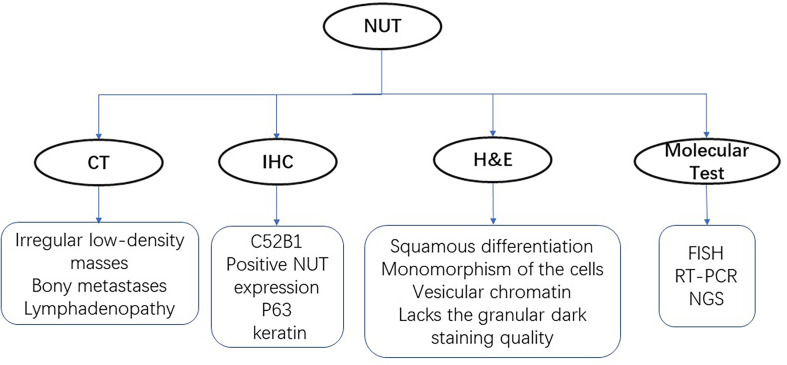
Diagnostic technologies for NUT-midline carcinoma. There are four main methods for identifying the NUT, including CT, H&E staining, immunohistochemistry (IHC), and molecular tests.

NUT-midline carcinoma can be diagnosed by testing a monoclonal antibody to NUT (C52B1) with immunohistochemical (IHC) nuclear staining (17, 18). However, mutation types of NUT rearrangements are unknown in a monoclonal antibody test. For targeted therapy, exact fusion partner gene should be identified by FISH, RT-PCR, and next-generation sequencing technology. Imaging examination of digital radiography (DR), computed tomography (CT) scans, and positron emission tomography (PET)/CT for patients are available methods for diagnosis, including head CT, 18F fluorodeoxyglucose (FDG) PET/CT, abdominal CT, and bone scintigraphy if needed (**Figure 1**). Chest CT scans were applied to assess the characteristics of lung lesion, involvement of the contralateral lung, lymphadenopathy, and pleural and osseous abnormalities. Other imaging examinations were used to identify the extrathoracic metastasis. CT imaging masses of primary lung NMC were mostly located in the center of the lung lobe, especially in the right lung and lower lobe. Primary pulmonary NMC usually showed large and irregular soft tissue masses with low density and fused with ipsilateral hilar and mediastinal lymph nodes. Patients with primary lung NMC often accompanied by pleural effusion, pleural thickening and obstructive atelectasis. Lymph node metastasis was seen in most patients, and the contralateral lung of patients was often not invaded. Extrathoracic metastasis of tumors was common, and bone is the most frequently metastatic site of the lung NMC. It was reported that one patient showed multiple bone metastases on CT and PET-CT, but the results of bone imaging were negative (19). This observation is consistent with other published studies, suggesting that negative bone imaging may not accurately exclude the bone metastasis of the tumor (20). In addition to bone, liver, breast, retroperitoneum, soft tissue, and adrenal glands are also important sites for NUT cancer metastasis. Imaging examination plays a significant role in the early diagnosis of pulmonary (NUT)-midline carcinoma.

Histologic appearances of NUT-midline carcinoma often overlap with many other poorly differentiated tumors. Differential diagnosis should be followed. Firstly, lymphoid epithelioid carcinoma has similar histological features; however, lymphoid epithelioid carcinoma often shows aggregates of cells, the margin is not clear and the nucleus often has prominent nucleoli, necrosis, and interstitial fibrous tissue hyperplasia are uncommon. Negative NUT antibody staining with positive expression of EBER fluorescent *in situ* hybridization are the most diagnostic characteristics. Secondly, the histology and morphology of pulmonary small cell carcinoma share some features with midline carcinoma. The immunohistochemical staining shows neuroendocrine biomarkers, high CD56 and TTF-1 expression, and positive NUT antibody staining. Thirdly, lymphocytic tumors are the most difficult to make differentiated diagnosis, because of its similar morphology, especially high-grade B-cell lymphoma. The tumor cells are usually scattered and diffusely arranged which is difficult to identify by immunohistochemical staining. The immunohistochemical staining would show negative NUT staining, LCA is positive, myeloid sarcoma biomarker CD 117 is positive, and CK staining is negative. In addition, primitive neuroectodermal tumor (PNET) should also be differentiated. The immunohistochemical staining of CD99 biomarker is diffusely positive, while CK is generally negative and NUT antibody biomarker is negative. FISH test can confirm the existence of EWS-FL1-1 gene fusion. Finally, the incidence of germ cell tumor (GCT) in the thoracic organs such as the mediastinum is relatively higher than upper respiratory track. The cell arrangement of NUT carcinoma and infiltration of interstitial lymphocytes are necessary to distinguish NMC from seminoma and embryonal cancer. Immunohistochemical staining assay of GCT would show negative CK staining and NUT focal nucleus (<5%), while NUT carcinoma is positive for CK and the nuclei is diffusely distributed (>50%).

**Figure 2:**
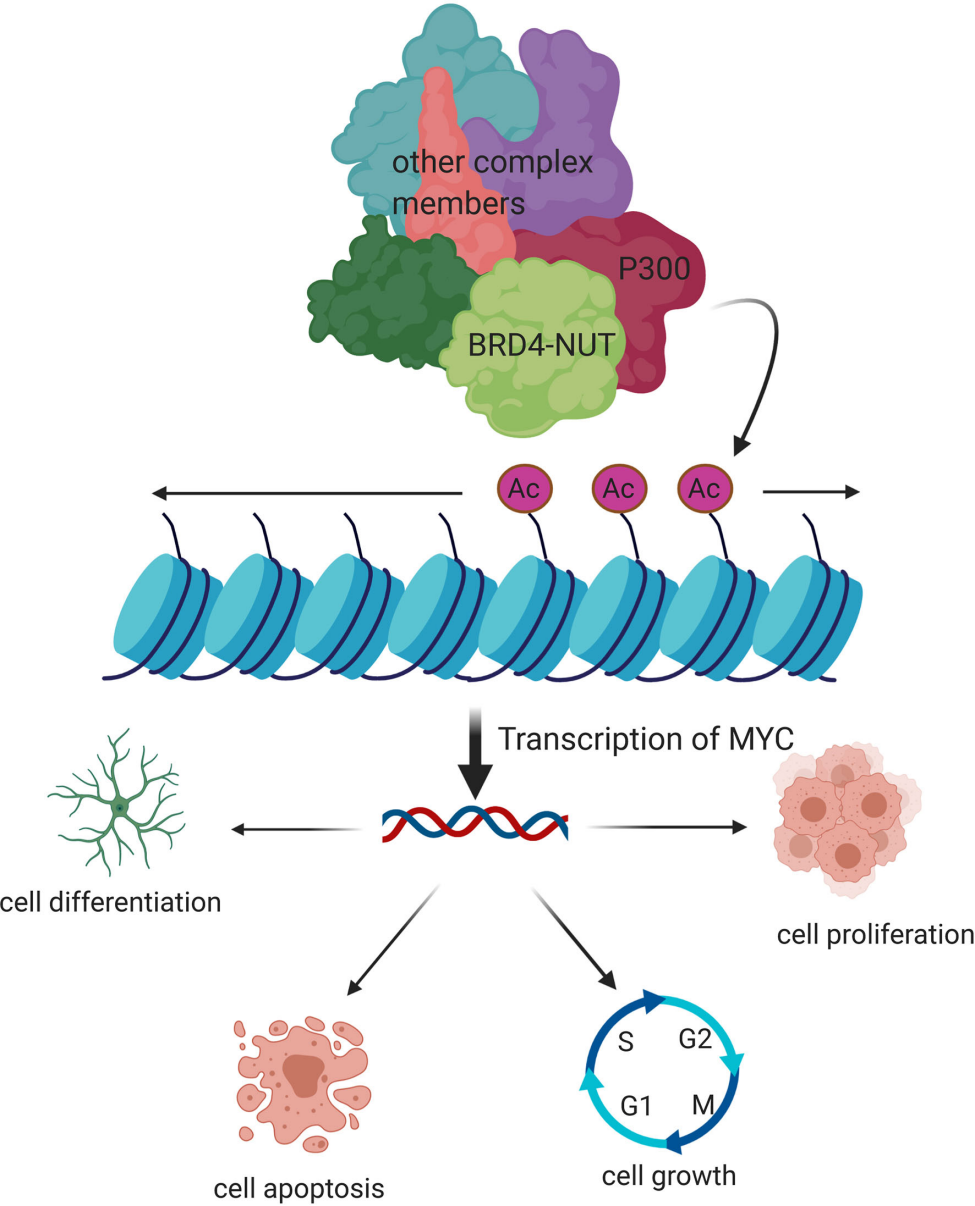
Molecular mechanism of pathogenesis of NUT-midline carcinoma. BRD4-NUT fusion oncoprotein, acetylated chromatin, and p300 form several huge regions that include the regulatory regions of MYC, p63, and MED24, which regulate cell transformation, differentiation, growth, and proliferation.

*NUTM1* gene with unknown function on chromosome 15 is usually expressed solely in testis tissue, and rearrangement of which is the main cause of NUT-midline carcinoma. BRD4 in BET family is the most common fusion partner gene, and BRD4-NUT fusion oncogene can be identified in two-thirds of the cases (21). Other relatively common fusion partner genes are NDS3 and BRD3. Moreover, MXD1, CIC, ZNF532, ZNF592, MXD4, BCORL1, and MGA have been reported recently (22–28). BRD4 is associated with many types of tumors and plays a crucial part in cell cycle regulation, transcriptional regulation, cell growth, and chromatin structure. Two key domains of BRD4 was retained in BRD4-NUT fusion oncoprotein. One is bromodomain which binds to acetylated histone, and the other is ET domain which binds to chromatin-modifying proteins. The acidic domain of NUT in fusion protein bound with histone acetyltransferase p300 can lead to histone acetylation. Furthermore, BRD4-NUT fusion oncoprotein, acetylated chromatin, and p300 form several huge regions that include the regulatory regions of MYC, p63, and MED24, which regulate cell transformation, differentiation, growth, and proliferation (**Figure 2**). Multiple studies have proved that p63 plays a key role in cell proliferation, survival, apoptosis, differentiation, cancer progression, and cancer metastasis. *MED24* gene, encoded as a transcriptional coactivator complex, is usually involved in gene expression. MYC, a transcription factor overexpressed in various types of cancer cells, has been proven to be a key inducer of many oncogenic pathways, such as the Wnt, mitogen-activated protein kinase (MAPK), and phosphatidylinositol 3-kinase (PI3K) signaling pathways (29–33). MYC gene regulates organ morphogenesis during embryogenesis and tissue repair. Wnt signaling is associated with various types of cancers such as colorectal, breast, and lung cancer. Compared with other signaling pathways, MAPK and PI3K pathways are involved in complex biological processes like cell proliferation, differentiation, transformation, apoptosis, and metabolism, which are closely associated with human diseases.

### Case Description: Primary Pulmonary NUT-Midline Carcinoma

**Figure 3:**
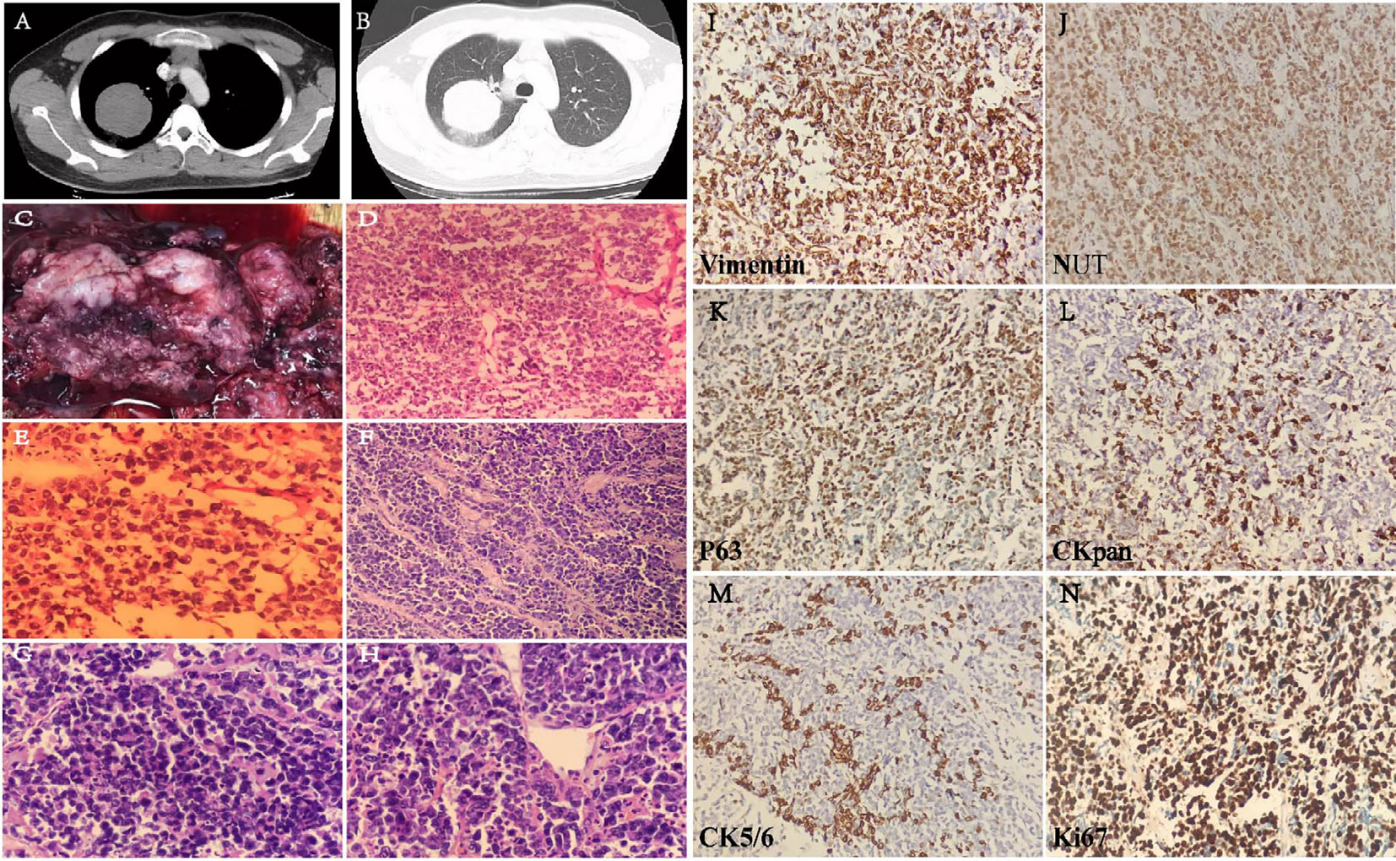
Lung CT image of the patient with soft tissue window **(A)** and lung window **(B)**. Gross sample and H&E images. **(C)** The gross image of pulmonary mass. **(D)** Frozen H&E section ×20. **(E)** Frozen H&E section ×40. **(F)** Paraffin H&E sections ×20. **(G, H)** Paraffin H&E sections ×40. Immunohistochemical staining **(I–N)** on six biomarkers. Vimetin (+), NUT (nuclear positive), P63 (mostly+), CK7 (few+), CK5/6 (few+), and Ki-67 index (80%).

A 16-year-old male patient presented with a pulmonary mass in the upper lobe of the right lung for 10 days. Lab examination results show NSE was high (16.93 ng/ml). Lung enhanced CT showed a round soft tissue density shadow in the upper lobe of the lung, the size was about 6.3 × 6.2 cm, and the edge is smooth. The enhanced CT scan showed the value of the mass was about 46 HU and the adjacent bronchus is compressed. The distal lung tissue showed strips of ground-glass opacity shadow (**Figures 3A, B**).

#### Surgical Findings

During the surgery, no adhesion was found in the thoracic cavity. The pleura was smooth and there was no significant effusion in the thoracic cavity. Pulmonary fissure developed normally. The tumor was located at the upper lobe of the right lung with a size of 6 × 5 cm. The tissue texture is hard and red. The mass was closed to the pleura. The tumor mass was relatively large, therefore, right upper lobectomy combined with lymph node dissection was performed.

#### Gross and Microscopic Pathological Findings

One lobe from lobectomy. A mass is seen in the lung. The size of the lung lobe is about 11 cm × 9 cm × 4.5 cm. The mass is located 0.6 cm away from the end of bronchial anastomosis, close to the lung capsule with a total size of 7 cm × 6 cm × 5 cm. The section surface is gray and gray-red, the texture is slightly brittle, the margin is not clear, bleeding, shows a cystic change, and easy to be broken. The gross image of pulmonary mass includes the following: the section is gray and gray-red, bleeding, and shows a cystic change (**Figure 3C**). Under the microscope, the tumor cells are extreme poorly differentiated, round, medium size, nested, or scattered. It is similar to the morphology of small cell carcinoma or lymphoid epithelioid carcinoma. Few fibrous connective tissues were seen between the cell nests. There are multiple foci and slice-like necrosis. The adhesion of tumor cells is relatively poor. Most nuclei is round or oval-round shape and medium size. The size of cells is about two to threefold over lymphocyte. The ratio of the nucleus over cytoplasm is high; the nuclei are irregular and atypical. The chromatin is fine or granular, vesicle shaped, and its nucleoli are prominent. Mitotic figures and apoptotic body are commonly seen; however, there is no significant “squamous differentiation” pattern in the stained slice. Focal infiltration of interstitial lymphocytes is seen and arrange in a flaky, scattered, or nested pattern (**Figures 3D–H**).

#### Immunohistochemical Staining

Immunohistochemical staining was performed on a broad spectrum of biomarkers (**Table 1**). In addition to NUT, several positively stained biomarkers have been identified. CKpan (partial+), NUT (nuclear positive), P63 (mostly+), TIF-1(few+), CK7(few+), CK5/P40(few+), CD5/6(few+), Syn (partial+), CD30 (few weak+), EMA (few weak+), Vimetin (+), CD99 (few weak+), HMB45, MelanA, CD3, CD20, CgA, CD31, CD34, Desmin, LCA, MyOD1, S-100, WT-1, PLAP, α-inhibin, CD138, CD38, MUM-1, TdT, ALK, Ki-67 index (80%), and florescence *in situ* hybridization EBEB (−). Some of the representative stainings are shown in **Figures 3I–N**.

**Table 1:** Immunohistochemical staining of biomarkers.

Antibody name	Expression level	Antibody name	Expression level
CKpan	Partial+	CD3	Negative
NUT	Nuclear positive	CD20	Negative
P63	Mostly+	CgA	Negative
TTF-1	Few+	CD31	Negative
CK7/6	Few+	CD34	Negative
CK5/P40	Few+	Desmin	Negative
CD56	Few+	LCA	Negative
Syn	Partial+	MyOD1	Negative
CD30	Few weak+	S-100	Negative
EMA	Few weak+	WT-1	Negative
Vimetin	+	PLAP	Negative
CD99	Few weak +	α-inhibin	Negative
HMB45	Negative	CD138	Negative
MelanA	Negative	CD38	Negative
TdT	Negative	MUM-1	Negative
ALK	Negative	Ki-67 index	Ki-67 index (80%)

#### Genomic Analysis of Primary Pulmonary NUT-Midline Carcinoma

Quantitative PCR was performed, and the result indicated that the patient was positive for NUT carcinoma. Dye-based qPCR was used to identify NUT gene product, and lung adenocarcinoma and water were used as reference. The design of amplification probes: forward probe is located in exon 10 and reverse probe is located in exon 2 of the *NUMT1* gene. The result showed that the CT value of sample is 26.34 which indicated a strong amplification. Moreover, the dissolution curve showed the product is specific. The CT value of reference sample is ≥34 which indicated a weak amplification. The dissolution curve showed nonspecific amplification which mostly was primer dimer. The results of Sanger sequencing showed positive for NUT rearrangement. The probe design: forward probe is located in exon 10 of the *BRD4* gene and reverse probe is located in exon 2 of the *NUMT1* gene. Amplification Sanger sequencing further confirmed that the translocation occurs between the *NUMT1* and *BRD4* genes (**Supplementary Figure S1**).

#### Related Tumor Signaling Pathway and Gene Annotation

Using the high-throughput gene sequencing technique, we have identified KIFB-related heterozygous mutations and gene changes that are associated with multiple tumor-related pathways (**Supplementary Table S1**). Some signaling pathways are associated with NMC. To be specific, most of the KIFB mutation leads to a dysfunction of those signaling pathways that are related to cell metabolism and differentiation. The dysfunction of these genes would lead to an increase in the risk of tumor development. Since NMC patients are often diagnosed in advanced stage, the surgical intervention is usually not an option. Thereby, biopsy sample from the surgery is relatively few. In 70% of the cases, translocation occurs with the juxtaposition of *NUT* (15q14) and *BRD4* (19p13.1) genes or *BRD3* (9q34.2, 6% of cases) and other unknown genes (24%). Usually, gene sequencing test was performed to evaluate the possible gene variants (**Supplementary Table S2**) in order to find possible gene mutations.

#### Follow-Up and Outcome

This patient was transferred to multiple places in Ji Nan (Shandong Province) and Beijing for medical consultation, and he received two traditional chemotherapies. The outcome of this case was unfortunately fatal, and the patient died 3 months after diagnosis.

## Discussion

Presently, less than 100 cases of NMC were reported worldwide. NMC can occur at any age; however, it has more likely been seen in young adults and children with no significant gender difference. NUT carcinoma is frequently diagnosed at advanced stage, thereby sample from surgical resection are relatively few. NUT carcinoma is characterized by *NUT* gene rearrangement. In 70% of the cases, the juxtaposition of *NUT* (15q14) and *BRD4* (19p13.1) genes, the *BRD3* (9q34.2, 6% of cases) and other unknown genes (24%), results in translocation products (34–36). The NUT carcinoma is generally misdiagnosed as squamous cell carcinomas (especially basal cell-like squamous cell carcinoma), undifferentiated carcinomas, small cell carcinoma, Aden squamous carcinoma, Ewing’s sarcoma or lymphoma, metastatic germ cell tumor, and acute lymphoma. The diagnosis of NMC is made by demonstration of NUT expression or NUT rearrangement by immunohistochemistry assay. NUT carcinoma is highly aggressive, and there is no effective therapy. The mean survival of NUT carcinoma is only 7 months (36, 37).

In NMCs, the primary tumor is mostly located in the upper digestive tract and mediastinum, and the lung NMC was very rare (38–40). The pathological characteristics of the tumor tissue are insignificant for diagnosis. Cases of NUT-midline carcinoma with cryptic translocation are likely to be overlooked. A 13-year-old girl once was misdiagnosed with germ cell tumor in earlier publication (41). Detailed examination plays a key role in the diagnosis and treatment of NUT-midline carcinoma.

Sustainable and effective treatment plans for NUT-midline carcinoma are still in exploration and research. Without standard therapeutic option for NMCs, this case in study received two traditional chemotherapies, while outcome of the case was not satisfactory (42, 43). According to previous literatures, radiotherapy and complete tumor resection is critical to improve progression-free survival (PFS) and overall survival (OS); however, chemotherapy has no benefit in OS outcome (21, 44). Once diagnosed as Ewing’s sarcoma, a NMC patient survived for 13 years after receiving local radiotherapy, while similar treatment strategies have no perceptible effect on other NMC patients (2). Although radiotherapy or chemotherapy can affect tumor progression within a brief period for some NMC patients, the average overall survival of patients is very short. As there are not enough clinical evidences, the prognostic effectiveness of surgery, radiotherapy, and chemotherapy is unclear.

Target therapy plays an increasingly important part in NMC treatment. Histone deacetylase inhibitors (HDACi) was reported to be a key inhibitor of tumor cell differentiation and growth (45). The response of a 10-year-old male NMC patient to HDACi vorinostat seemed good after 5 weeks of treatment. Because of severe nausea and vomiting, the therapy was forced to terminate. Unfortunately, the tumor continued to grow and deteriorated, and the boy died 11 months after diagnosis (46). As significant members in the bromodomain and extraterminal motif (BET) protein family, BRD3 and BRD4 are the most important fusion partners of NUTM1. A serial of anticancer compounds that targeted BTE were developed. Bromodomain inhibitors (BETis) can directly bind to the bromodomain and target the BRD3/4-NUTM1 fusion proteins expressed in tumor tissue. Efficacy of BETi was proved to be better in NMC cell lines with BRD4-NUTM1 ex11:ex2 fusion than those with BRD4-NUTM1 ex15:ex2 or ex14:ex2 fusions (47). Although HDACi and BETi may dramatically prolong OS of NMC patients, toxicity, side effect, and acquisition of these inhibitors are major challenges that cannot be underestimated (48, 49).

As a rare and aggressive malignant tumor, NUT-midline carcinoma can be identified by combining PET/CT, H&E staining, IHC, and molecular tests. The development of NUT carcinoma might be associated with MYC, p63, and MED24 and the Wnt, MAPK, and PI3K signaling pathways. Our study provided a detailed molecular mechanistic review on NMC and established a procedure to identify pulmonary NMC. Early identification, timely symptomatic treatment, and progressive targeted treatment for NMC patients are an extremely urgent need.

## Data Availability Statement

The original contributions presented in the study are included in the article/**Supplementary Material**. Further inquiries can be directed to the corresponding authors.

## Ethics Statement

The studies involving human participants were reviewed and approved by the Weifang People’s Hospital. The patients/participants provided their written informed consent to participate in this study.

## Author Contributions

WL, XL, and GFZ conceived and designed the experiments. YZ, XD, LL, QH, GYZ, and KH performed the experiments. YZ and TL analyzed the data. YZ wrote the first draft. All authors contributed to the article and approved the submitted version.

## Funding

This work was supported by the Project of Key R&D Program of Shandong Province (2015GSF118168), the Project of Soft Science & Development program of Weifang Bureau of Science and Technology (2019RKX039), and the Project of Medical and Health Technology Development of Shandong Province (2017WS807).

## Conflict of Interest

TL is an employee of the Qingdao Geneis Institute of Big Data Mining and Precision Medicine.

The remaining authors declare that the research was conducted in the absence of any commercial or financial relationships that could be construed as a potential conflict of interest.

## Publisher’s Note

All claims expressed in this article are solely those of the authors and do not necessarily represent those of their affiliated organizations, or those of the publisher, the editors and the reviewers. Any product that may be evaluated in this article, or claim that may be made by its manufacturer, is not guaranteed or endorsed by the publisher.
