# Supplementary material for: Case Report and Literature Review: Primary Pulmonary NUT-Midline Carcinoma
Source: Front Oncol. 2021 Aug 30;11:700781. doi: 10.3389/fonc.2021.700781 (PMC8435908; doi:10.3389/fonc.2021.700781)
Supplement: Supplementary file 1 [file DataSheet_1.docx]

**Primary pulmonary NUT-midline carcinoma: a case report and literature review**

Yunxiang Zhang^1^, Kai Han^1^, Xiaotong Dong^1^, Qian Hou^1^, Tianbao Li^2^, Li Li^3^, Gengyin Zhou^3^, Xia Liu^4,*^, Guifeng Zhao^5,*^ Wei Li^6^*

^1^ Pathology Department, Weifang People’s Hospital, Weifang 261014, P. R. China

^2^ Qingdao Geneis Institute of Big Data Mining and Precision Medicine, Qingdao 266000, P. R. China

^3^ Pathology Department, Qilu Hospital of Shandong University, Jinan 250012, P. R. China

^4^Ophthalmology Department, Affiliated Hospital of Weifang Medical University, Weifang 261031, P. R. China

^5^Prenatal diagnosis Department, Weifang People’s Hospital. Weifang 261014, P. R. China

^6^Thoracic Surgery Department, Weifang People’s Hospital, Weifang 261014, P. R. China

*Corresponding authors:

Wei Li: wfliwei0213@163.com

Guifeng Zhao: wfrmzgf @126.com

Xia Liu: liuxia1978@163.com

**Supplements**


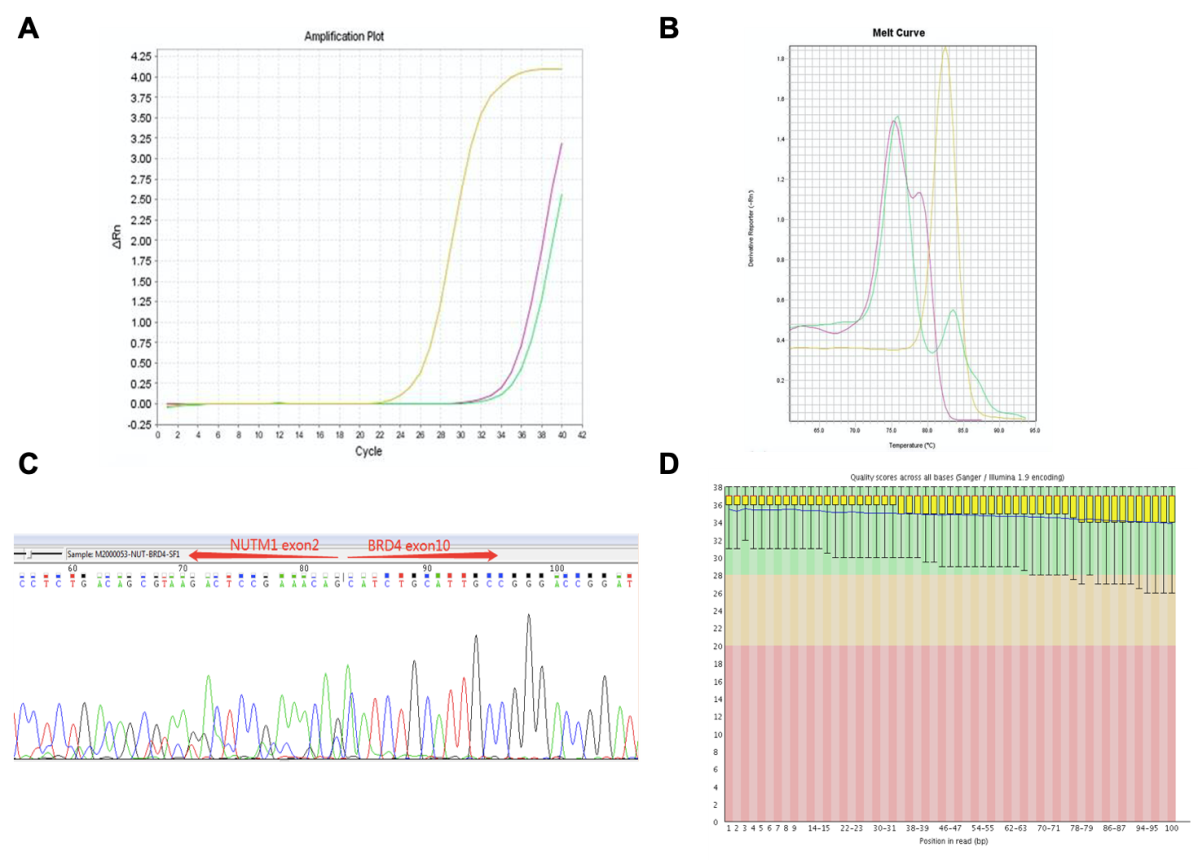


**Figure S1**. (A) Amplification and (B) dissolution curve, NTC (pink curve) represented negative control, green curve represented amplification product of pulmonary adenocarcinoma, NUT carcinoma (yellow curve) represented NUT specific amplification product of NUT gene. (C) Sanger sequencing results. (D) The quality control of high-throughput sequencing.

**Table S1 Gene tests related to tumor signaling pathways**

| Tumor-related signaling pathway | Gene mutation |
| --- | --- |
| MAP kinase signal pathway | HNF1A |
| SAPK/JNK signaling cascades | HNF1A |
| Receptor tyrosine kinase signaling pathway | ERBB4 |
| Cell cycle signaling pathway | RAD50, MSH6, BLM |
| Immunology signaling pathway | BCL10 |
| DNA Damage/repair signaling pathway | CHD2, RAD50, MSH6, BLM, BRCA2, FANCC |
| Cellular metabolism signaling way | PRSS1, HNF1A |
| Stem Cell Markers and differentiation | HNF1A |
| Wnt signaling pathway | HNF1A |
| Notch signaling pathway | HNF1A |
| Epigenetic changes signaling pathway | CHD2, DICE1 |

**Table S2 Genes involving tumor-related signaling pathway**

| **Gene Name** | **Mutation Information** | **Freq.** | **Gene interpretation** |
| --- | --- | --- | --- |
| BLM | NM_000057  c.1536dupA p.G512fs | 1.47% | BLM RecQ like helicase. BLM is a DNA helicase, plays an important role in maintaining genome integrity ^[8]^. BLM malfunction can lead to bloom syndrome and increase the risk of cancer ^[9]^. This variant is not documented in Clinvar, and clinical significance and biological function cannot be identified |
| BRCA2 | NM_000059  c.1806delA  p.I605Yfs*9 | 1.24% | Breast cancer susceptibility2 is a tumor suppressor that participates in the homologous recombination pathway of double –stranded DNA repair, thereby playing a role in maintaining genomic stability ^[10]^ it causes a frameshift mutation, which may lead to protein dysfunction. It is a pathogenic mutation. |
| CHD2 | NM_001271  c.4165dupA  p.M1388fs | 4.31% | Chromodomain helicase DNA Binding Protein2 (CHD2) is a chromatin remodeling protein that regulates gene expression and plays a role in neuronal development ^[11, 12]^. CHD2 mutation is associated with chronic lymphocytic leukemia ^[13]^. This variant is not documented in Clinvar, and clinical significance and biological function cannot be identified. |
| CHD2 | NM_001271  c.3725delA  p.E1242fs | 2.94% | Chromodomain helicase DNA Binding Protein2 (CHD2) is a chromatin remodeling protein that regulates gene expression and plays a role in neuronal development ^[14,15]^. CHD2 mutation is associated with chronic lymphocytic leukemia ^[16]^. This variant is not documented in Clinvar, and clinical significance and biological function cannot be identified. |
| DICER1 | NM_001195573  c.1630C＞T  p.R544X | 2.02% | DICER1gene encodes a protein with a dexh box at the amino end and an RNA motif at the carboxyl end. DICER is involved in microRNA synthesis and cell proliferation ^[17]^. Dysfunction of dicer1 is associated with multiple cancers, including endometrial cancer, pleural lung blastoma (PPB) cystic nephroma (CN) and sertolileydig cell tumor (SLCT), This mutation cause protein translation terminates prematurely, cause results in the loss of function of translated protein. It is a pathogenic mutation in clinvar. |
| ERBB4 | NM_001042599  c.1177C＞T  p.R393W | 1.81% | Receptor tryroine-protein kinase erbB-4, also called HER4. It encodes a member of the ERMM family which plays a role in cell survival and differentiation. The active mutation of ERBB4 is commonly seen in melanoma and also can be found in pulmonary adenocarcinoma. ERBB4R393W is located in the extracellular structure domain of EBBB4 (Uniprot.org). R393W has been reported in the literature, however, there is no biochemical features, thereby, its effect of Erbb4 remains unclear ^[18]^. |
| FANCC | NM_000136  c.520C＞T  p.R174X | 1.08% | Fanconi anemia entation group C is a member of Fanconi anemia code group. It participates in DNA repairs. Gerline FANCC mutations associated with Fanconi anemia, which increases susceptibility to multiple cancers, incluing pancreatic cancer and colorectal cancer. This mutation is recorded as pathogenic mutation in Clinar database ^[19]^. |
| HNF1A | NM_000545  c.865dupC  p.G288fs | 1.29% | Hypoxia inducible factor 1 alpha subunit is transcription factor with cancer suppressing activity, it plays hepatocyte differentiation and glucose transportation. Dysfunction and mutation of HNF1A is associated with hepatic adenoma. HNF1AG288does not belong to any of known HNF1 functional protein domain (Uniprot.org). It is not documented in Clinvar, thereby its effect on HNF1Aprotein is remained unclear ^[20]^. |
| MSH6 | NM_000179  c.3254delC  p.T1085fs | 1.67% | MutS homolog 6 encodes a member of the DNA mutation repair muts family. MSH6 combines with MSH2 to form mutS-alpha complex ^[21]^ and is associated with microsatellite instability (MSI) ^[22]^. MSH^ T1085 fs amino acid position 1085has a frameshift mutation which leads to early termination of translation and production of truncated protein (Uniprot.org). T1085fs has been reported ^[23]^. However, no further studies been conducted. Therefore, its effect of msh6 protein remains unclear. |
| PRSS1 | NM_002769  c.86A＞T  p.N29I | 22.44% | Serine protease 1 is a trypsinogen which involved in peptide cleavage ^[24]^. Germline PRSS1 mutation is association with familial pancreatitis, increase the risk of pancreatic cancer. this mutation is interpreted as a pathogenic mutation in Clinvar, which might be related to familial pancreatitis. |
| RAD50 | NM_005732  c.2157delA  p.L719fs | 2.82% | RAD50 Double Strand Break repair protein: RAD50 combines Mre 11 Nbs1 to form complex, and repair broken double strands, mediate DNA recombination, and maintains telomere length ^[25]^. This mutation can be found in Burkitt lymphoma, myeloid leukemia, ovarian cancer, liver cancer, and endometrial cancer ^[26-28]^ this variation is not recorded in Clinivar since its biological function is unclear. |
