# Supplementary figures and images for: Case Report and Literature Review: Primary Pulmonary NUT-Midline Carcinoma
Source: Front Oncol. 2021 Aug 30;11:700781. doi: 10.3389/fonc.2021.700781 (PMC8435908; doi:10.3389/fonc.2021.700781)

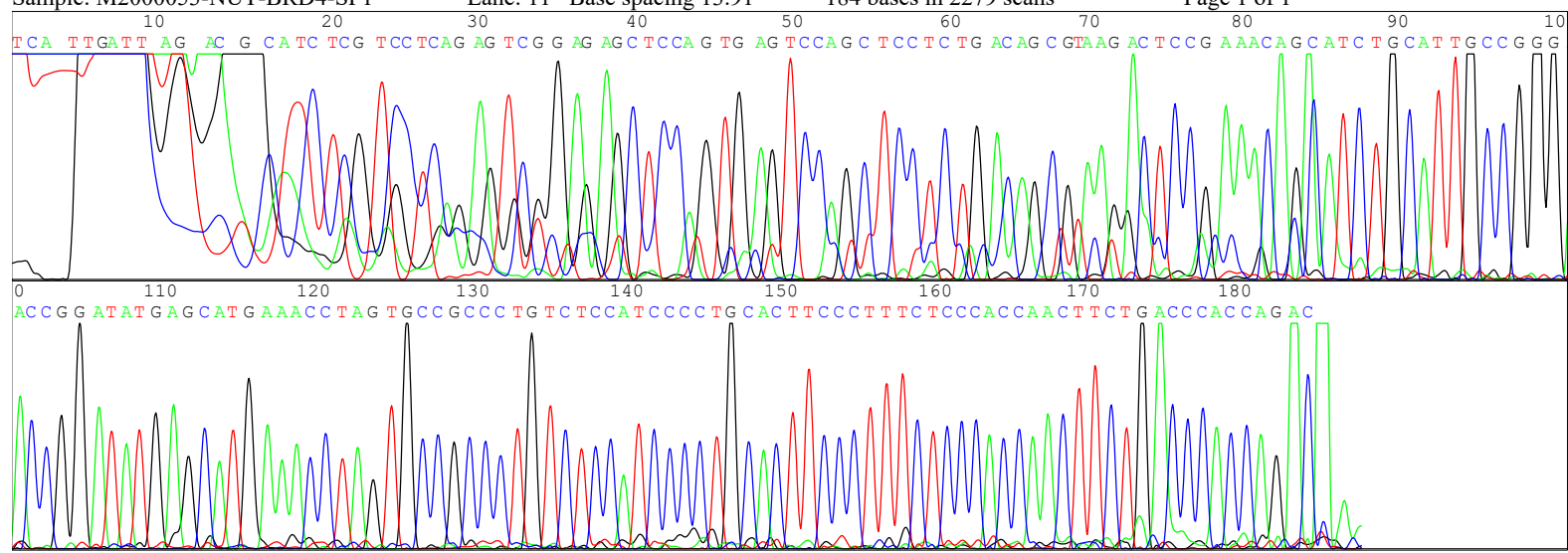

Supplement: Supplementary file 2 [file DataSheet_2.pdf]

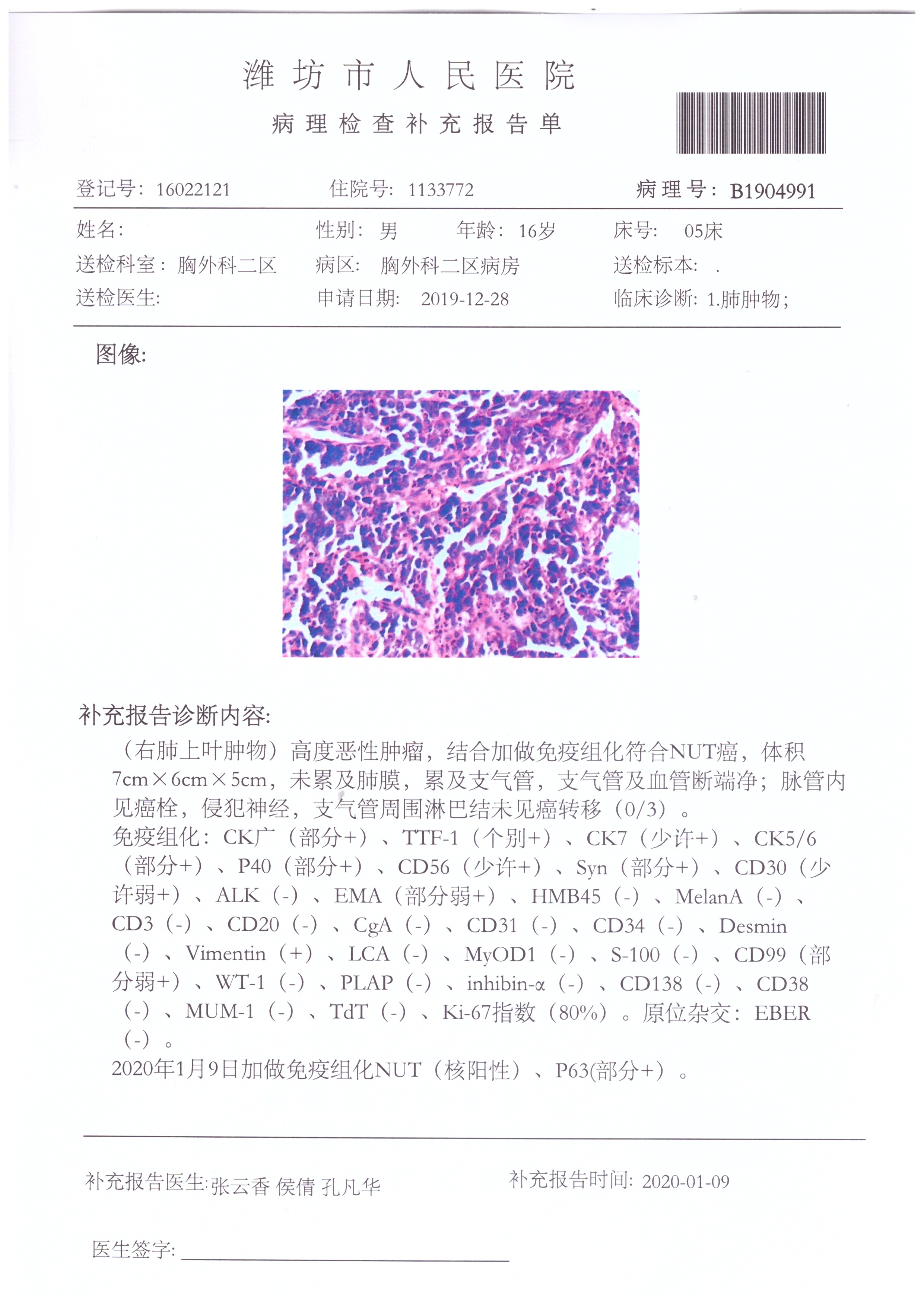

Supplement: Supplementary file 4 [file Image_1.jpeg]

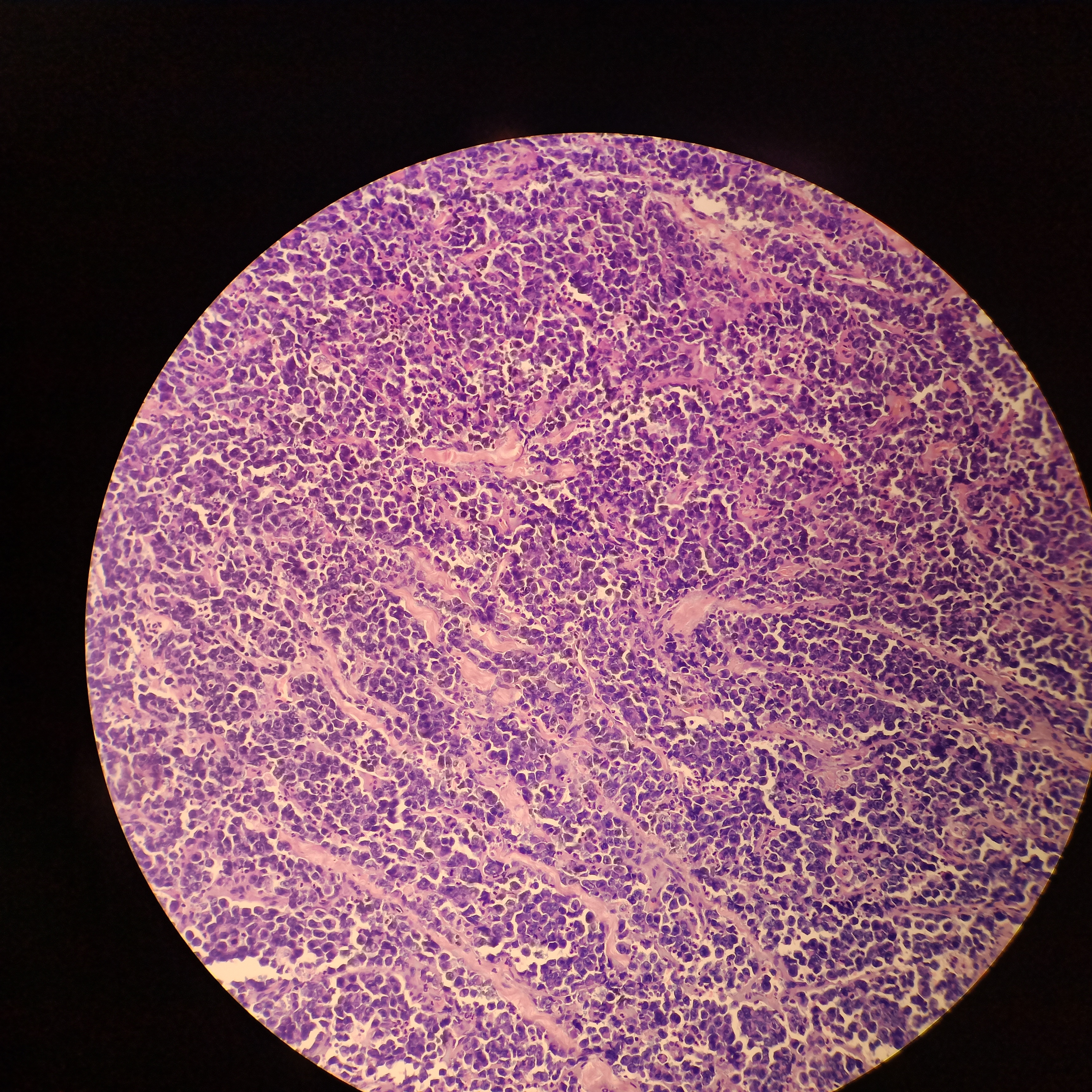

Supplement: Supplementary file 5 [file Image_2.jpeg]

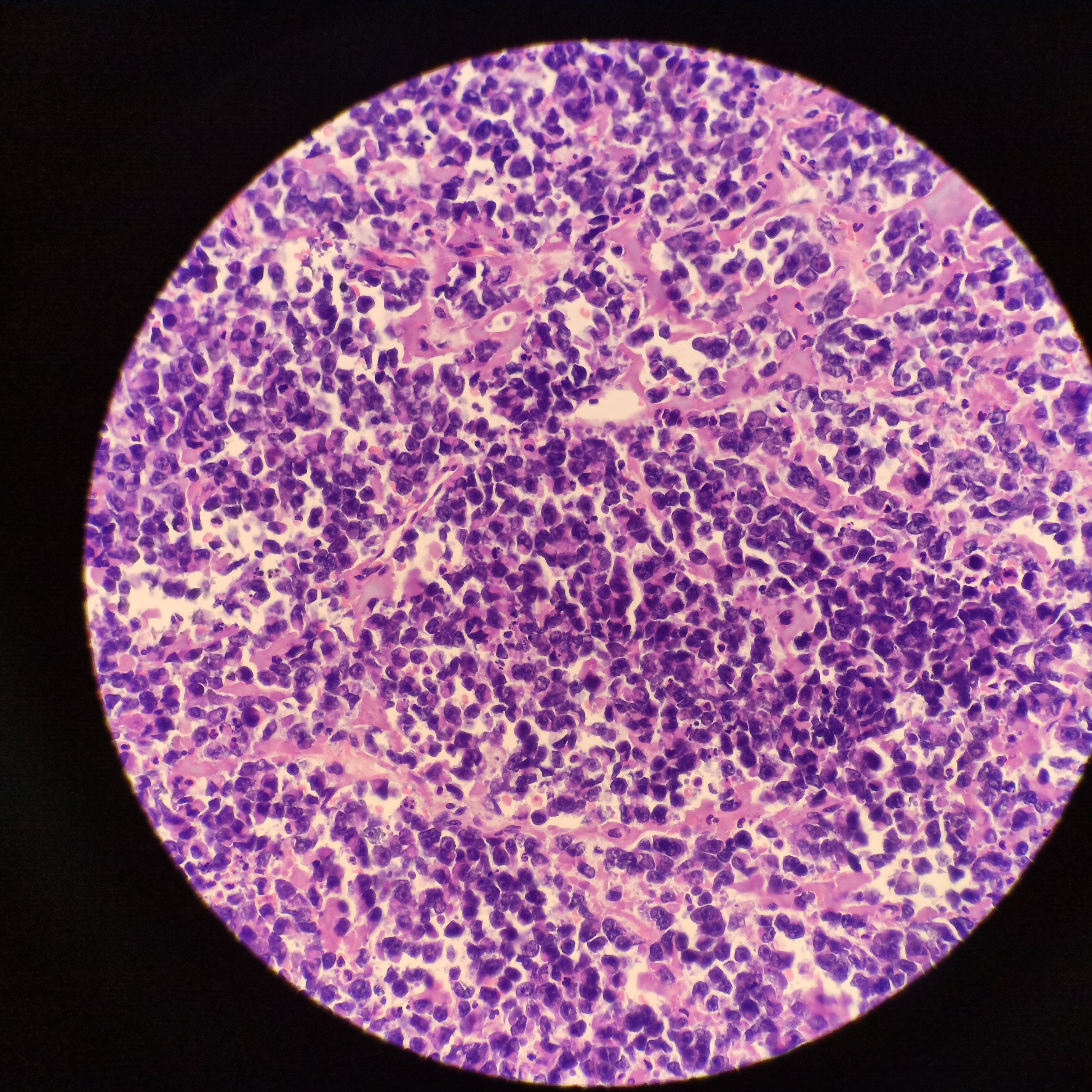

Supplement: Supplementary file 6 [file Image_3.jpeg]

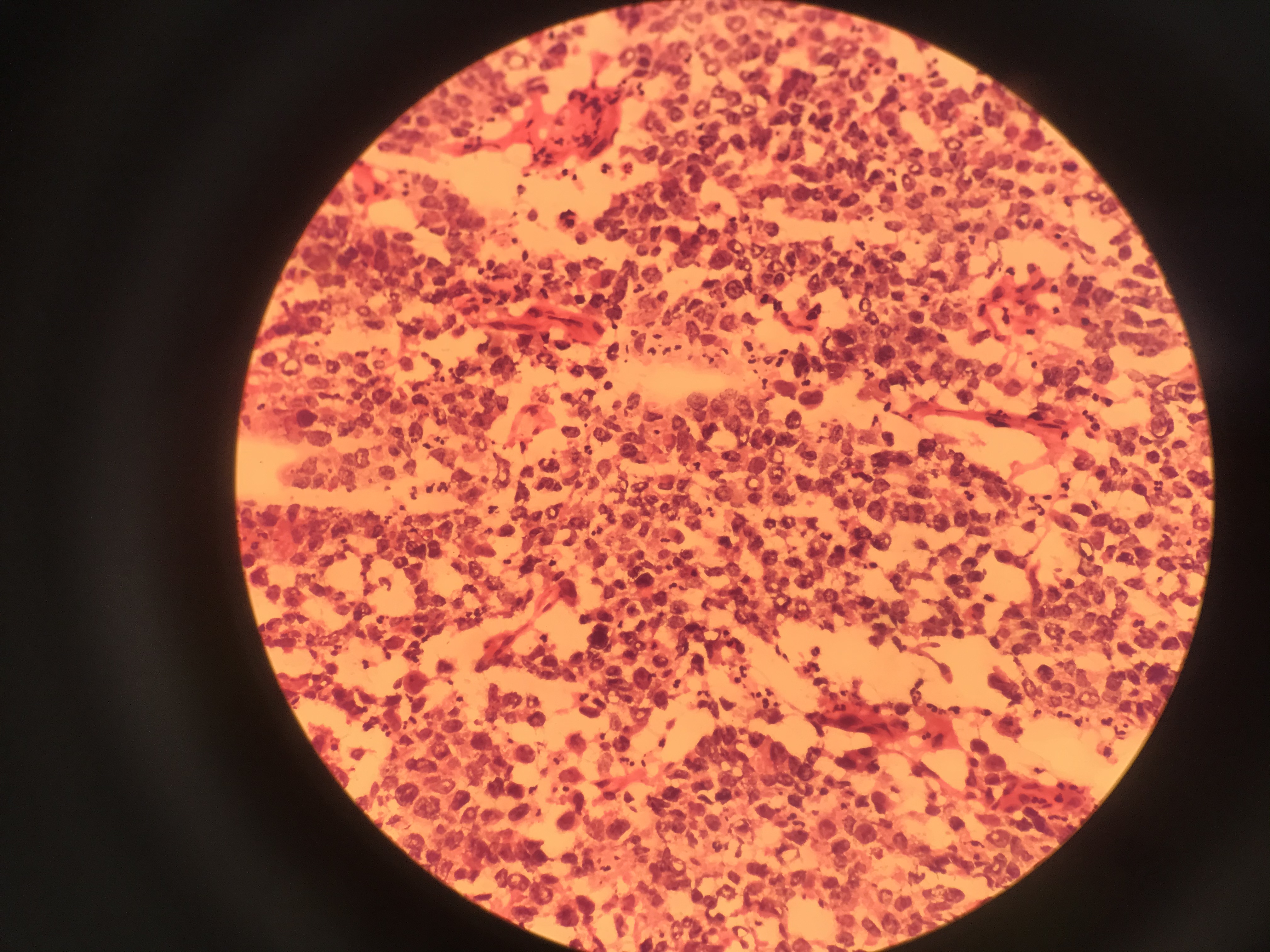

Supplement: Supplementary file 7 [file Image_4.jpeg]

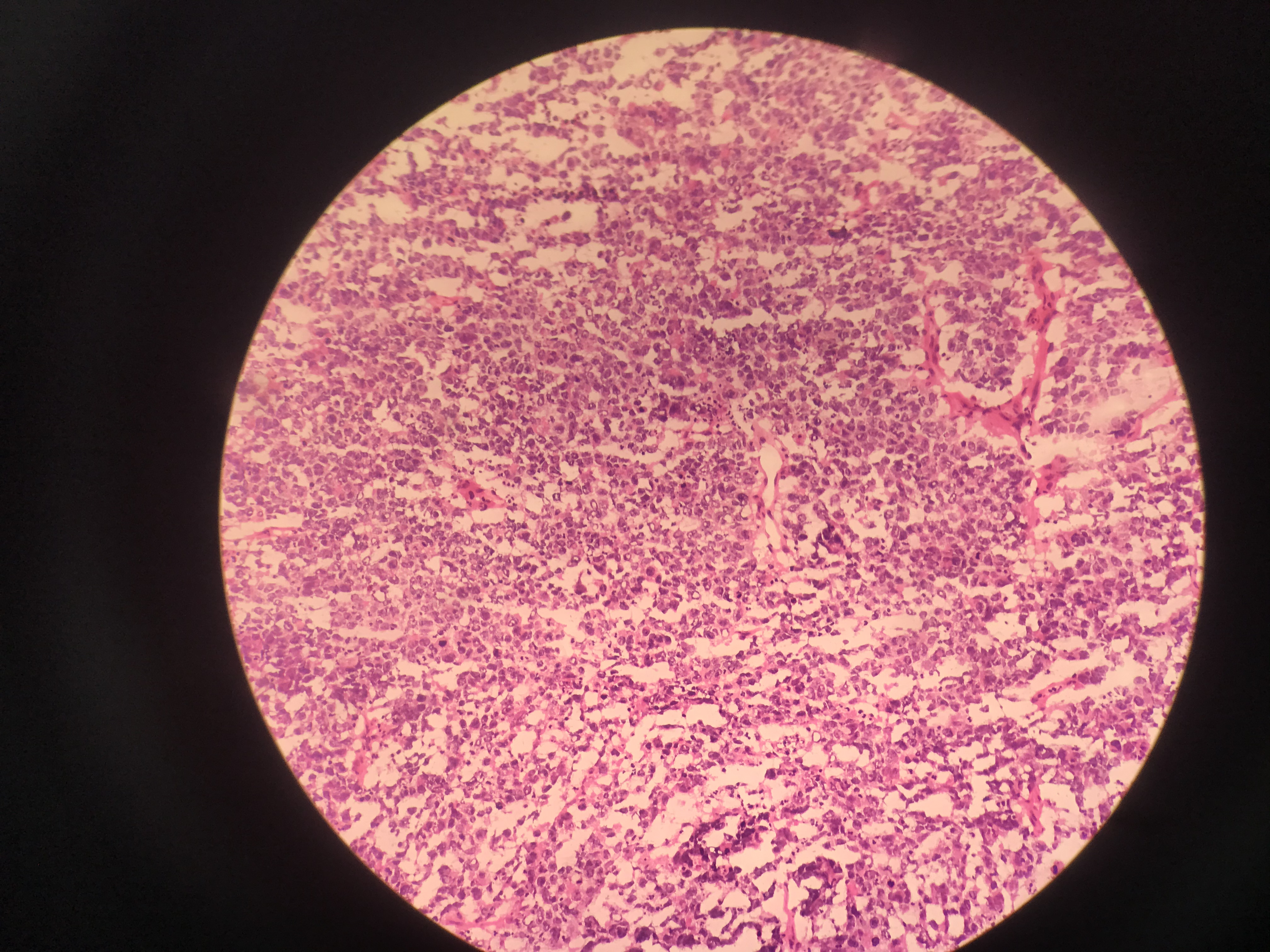

Supplement: Supplementary file 8 [file Image_5.jpeg]

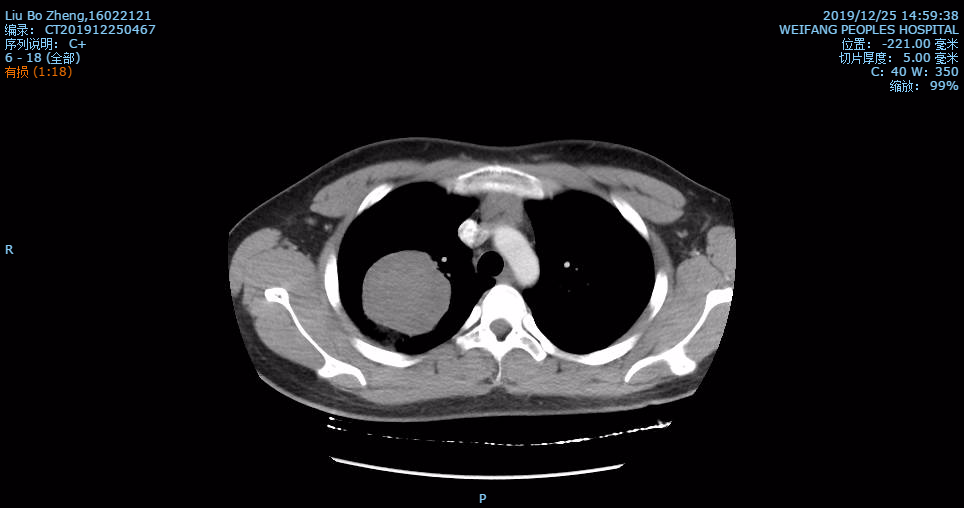

Supplement: Supplementary file 9 [file Image_6.png]

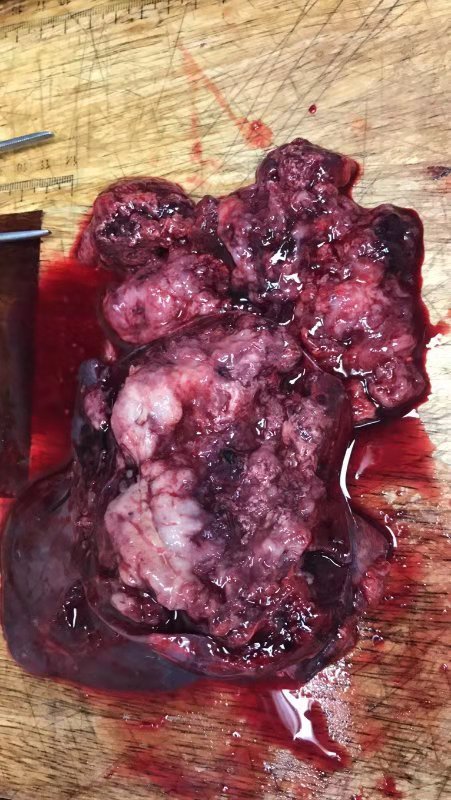

Supplement: Supplementary file 10 [file Image_7.jpeg]

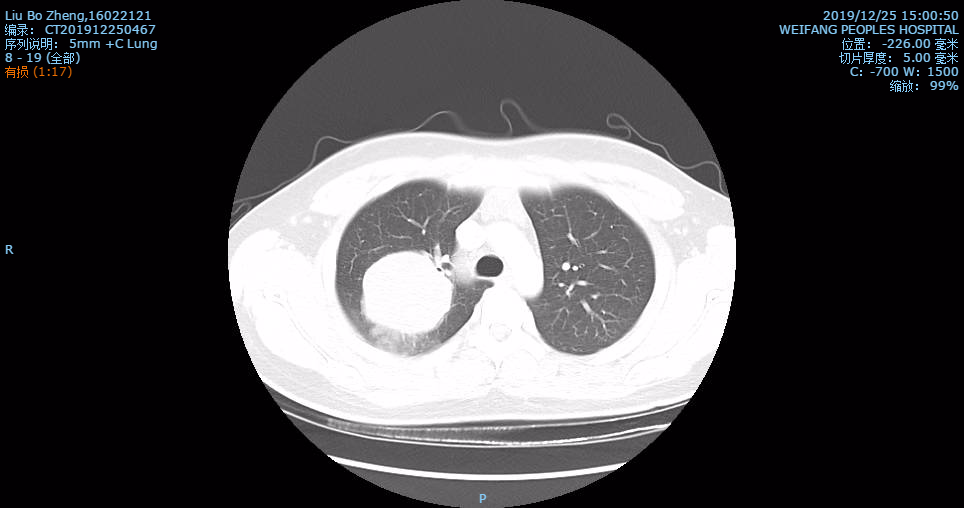

Supplement: Supplementary file 11 [file Image_8.png]

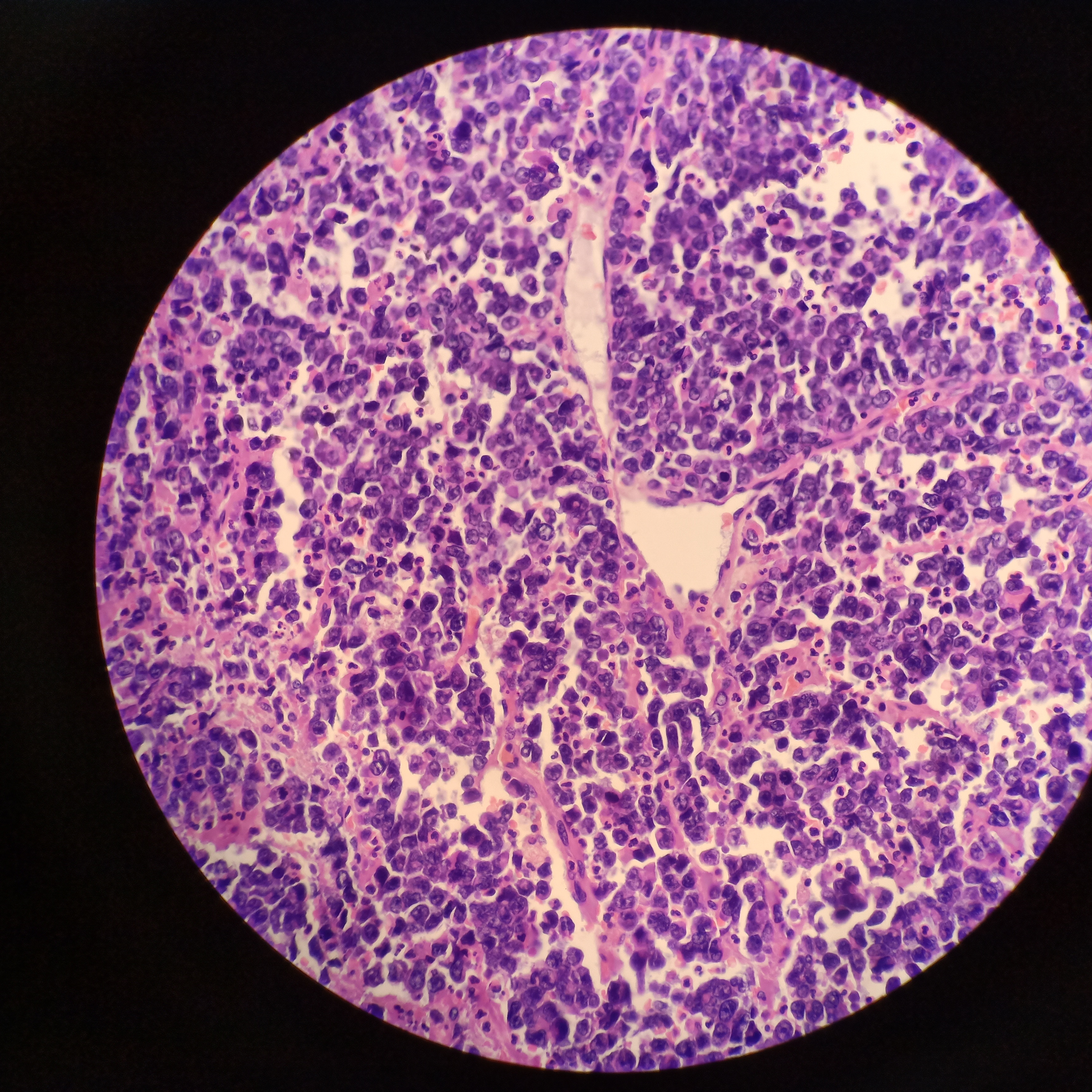

Supplement: Supplementary file 12 [file Image_9.jpeg]

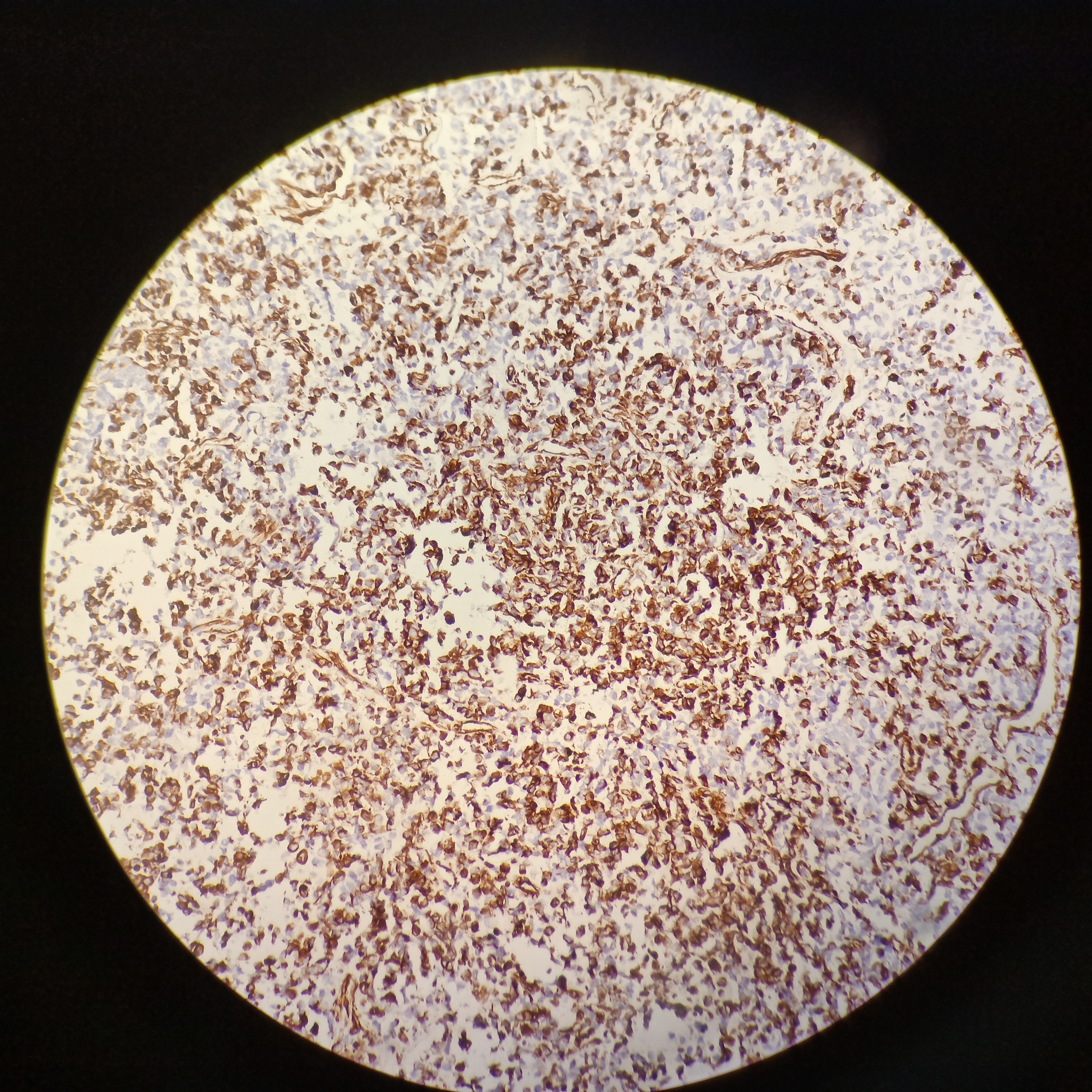

Supplement: Supplementary file 13 [file Image_10.jpeg]

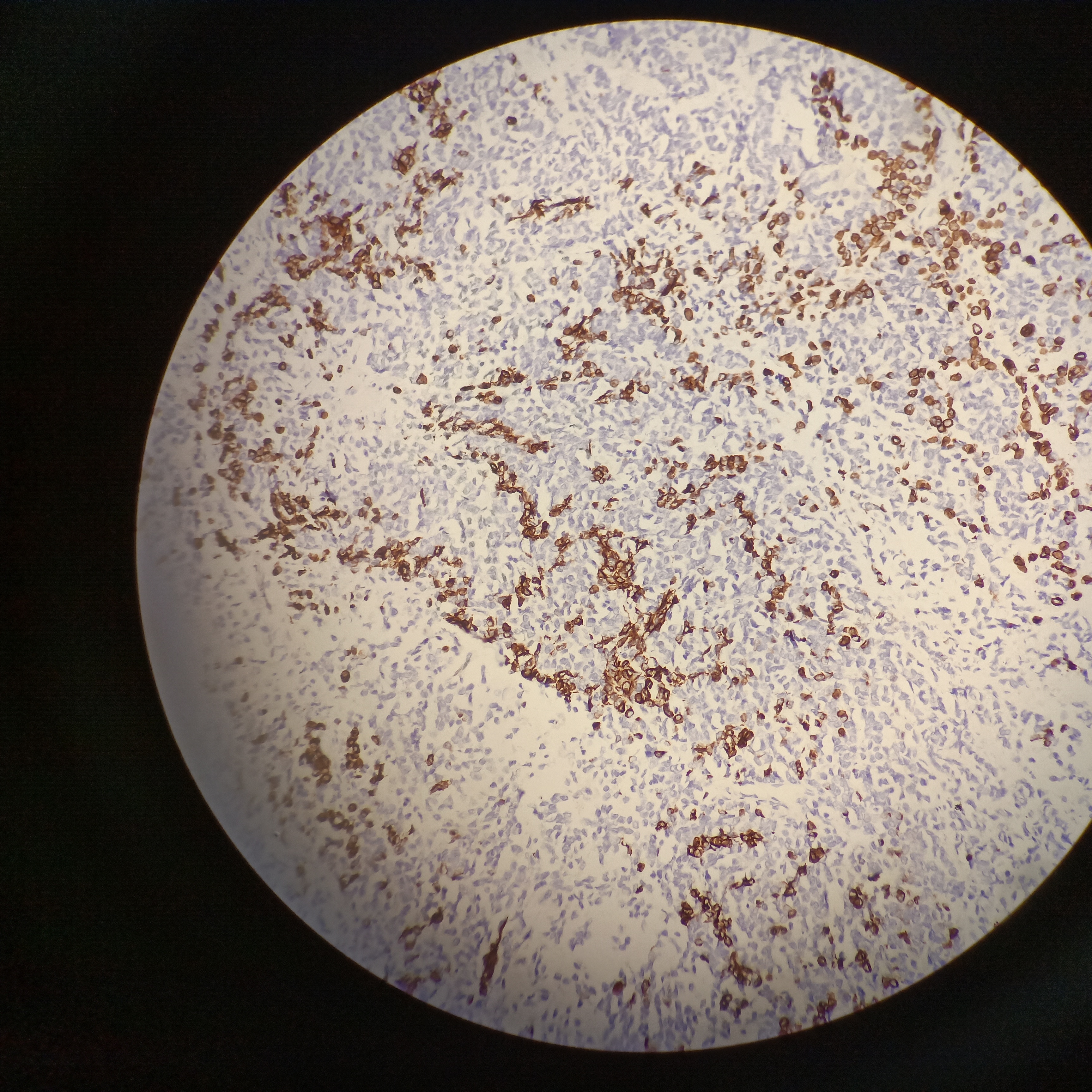

Supplement: Supplementary file 14 [file Image_11.jpeg]

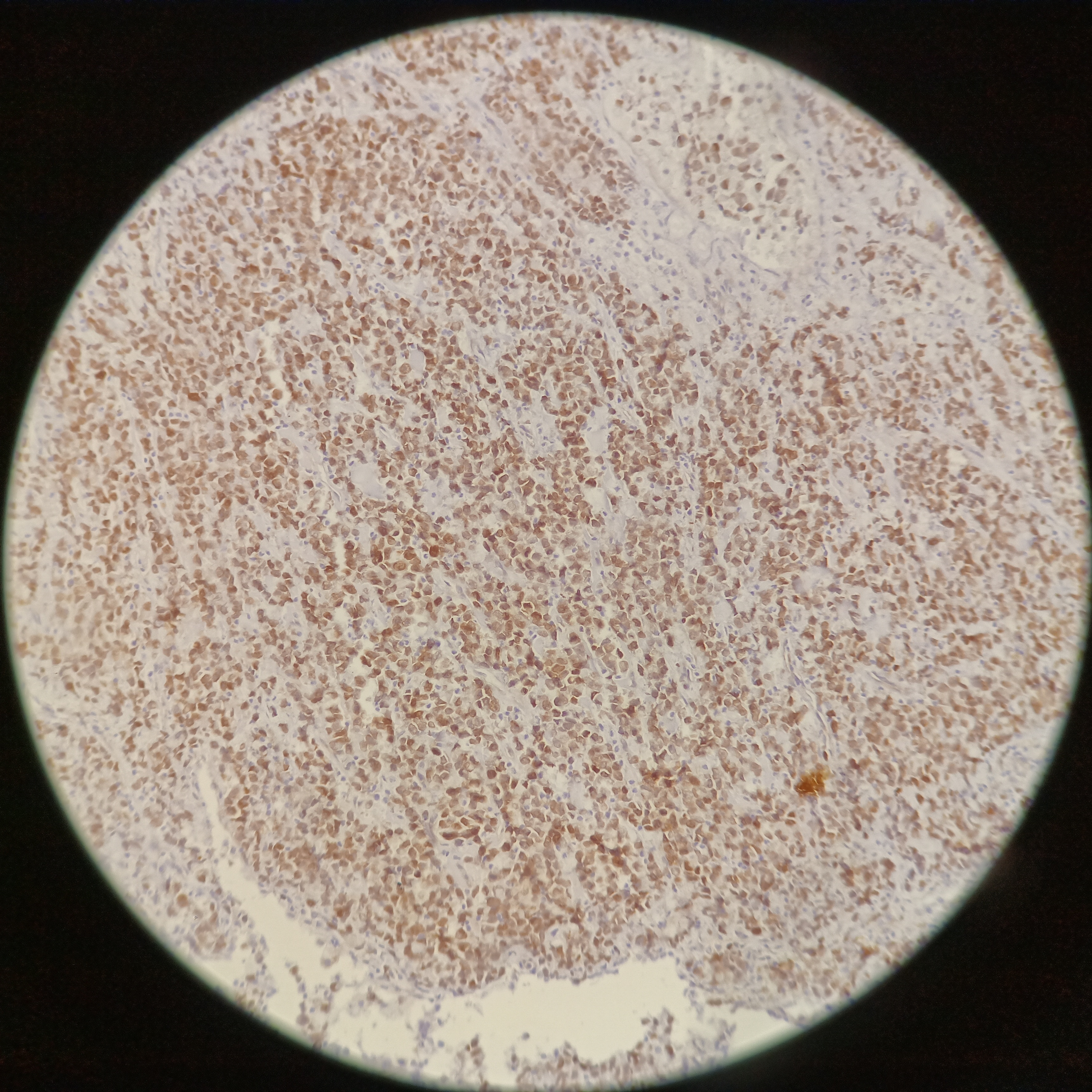

Supplement: Supplementary file 15 [file Image_12.jpeg]

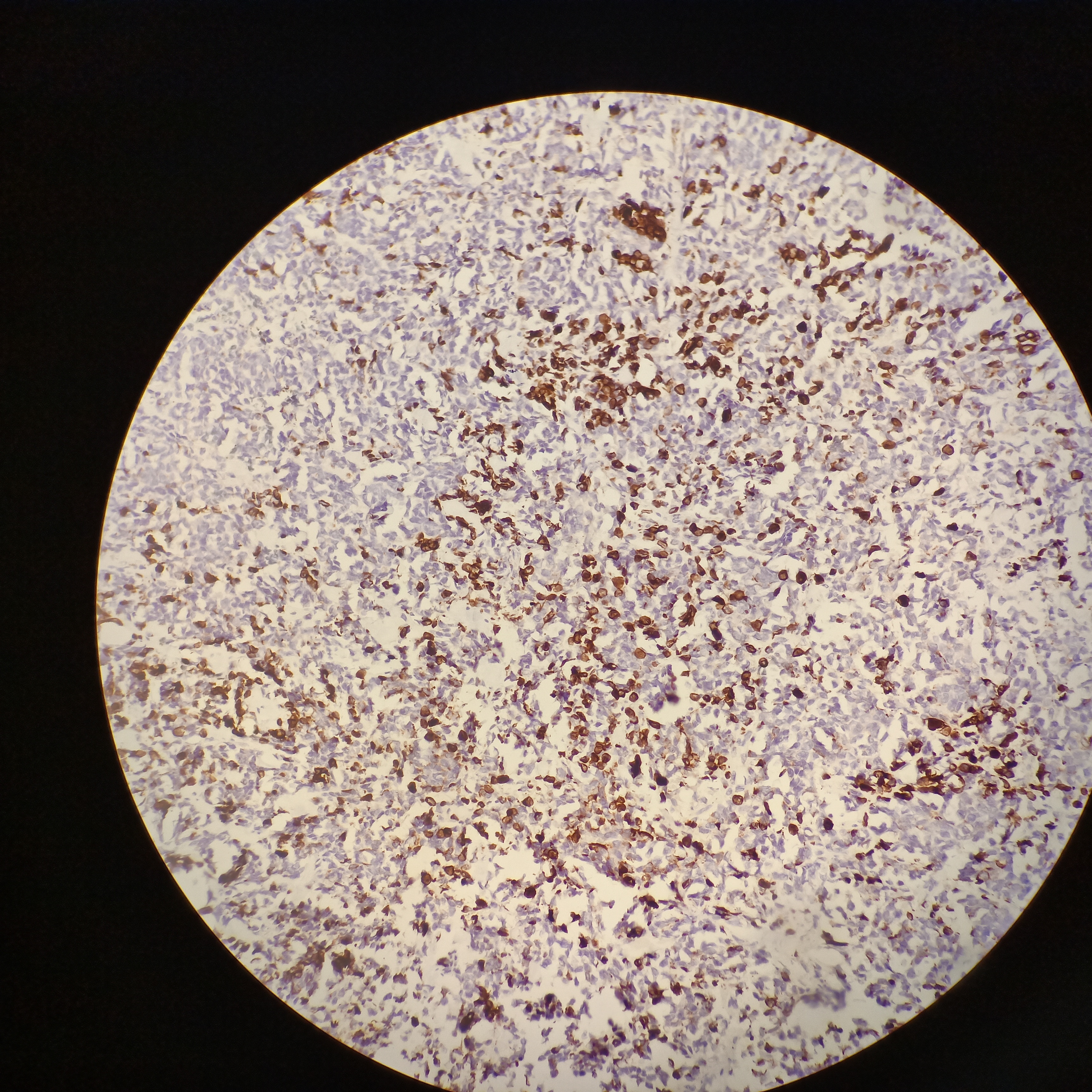

Supplement: Supplementary file 16 [file Image_13.jpeg]

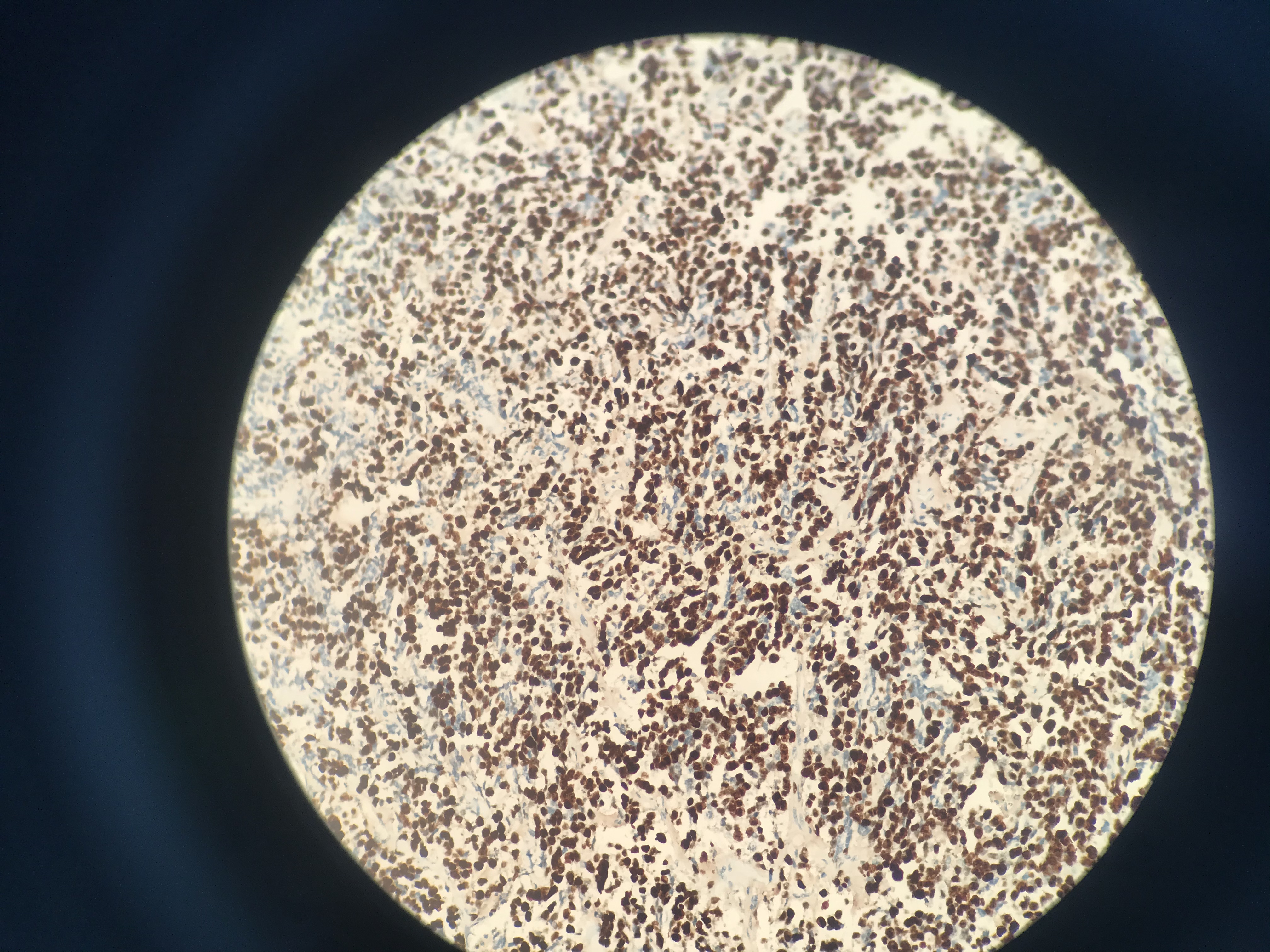

Supplement: Supplementary file 17 [file Image_14.jpeg]

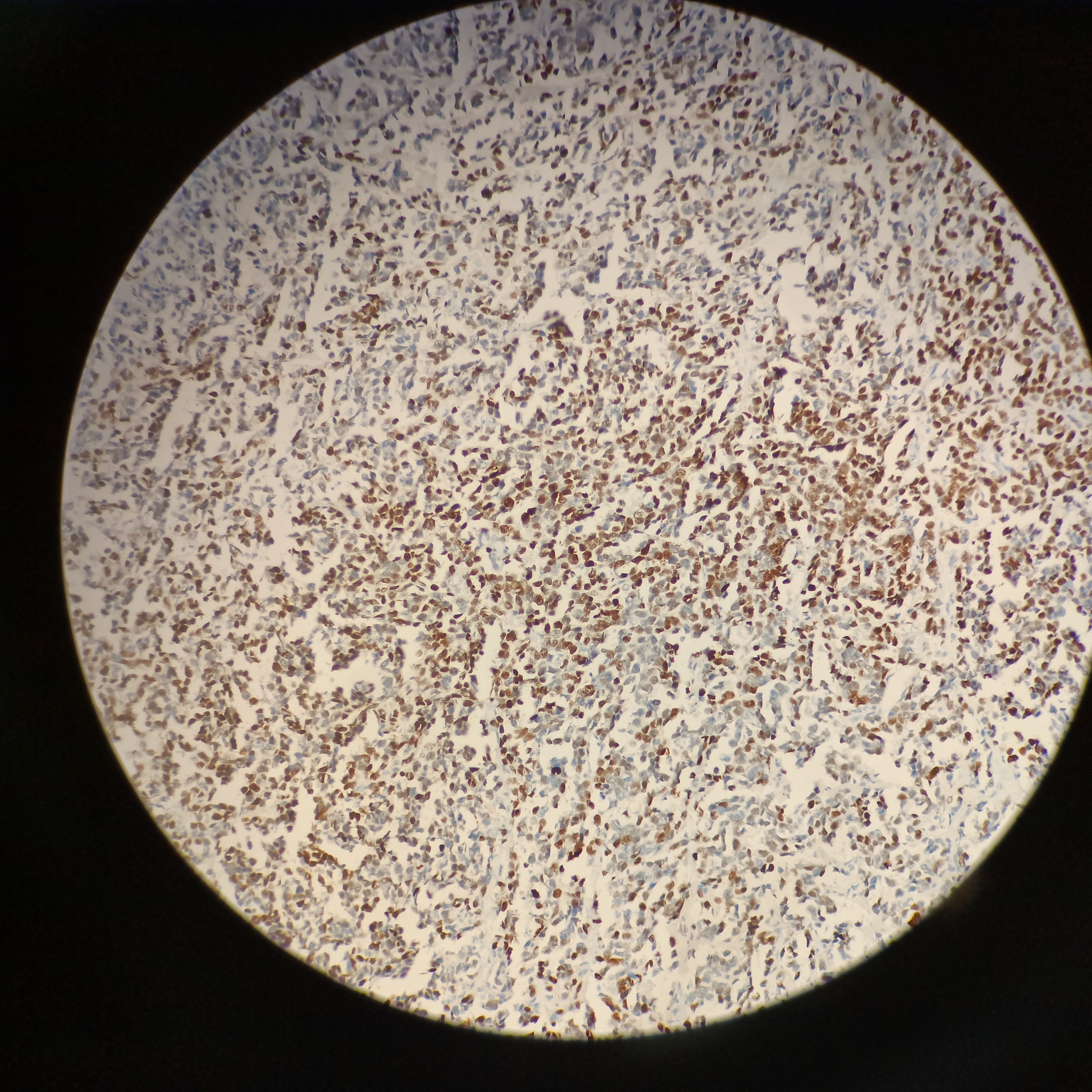

Supplement: Supplementary file 18 [file Image_15.jpeg]
